# Variability of predictive markers (hormone receptors, Her2, Ki67) and intrinsic subtypes of breast cancer in four consecutive years 2015–2018

**DOI:** 10.1007/s00432-019-03057-0

**Published:** 2019-10-18

**Authors:** Lidija Stevanovic, Matthias Choschzick, Linda Moskovszky, Zsuzsanna Varga

**Affiliations:** Institute of Pathology and Molecular Pathology, University Hospital Zurich, Schmelzbergstrasse 12, 8091 Zurich, Switzerland

**Keywords:** Breast cancer, Hormone receptors, Her2, Ki67 Index, Intrinsic subtypes

## Abstract

**Purpose:**

Accurate monitoring of predictive markers is of utmost importance as oncological treatment decisions almost entirely depend on these factors. In this study, we conducted a quality control assessment on hormone receptors, Her2 status, Ki67 Labelling Index (LI) and histological grading in breast cancer over 4 years (2015–2018).

**Methods:**

Altogether 2214 consecutive breast cancer cases were included. Data on estrogen (ER) and progesterone receptors (PR), Her2 and Ki67, were available in all cases and were tested mostly on preoperative biopsies, in selected cases on postoperative surgical specimens. ER, PR, and Ki67 were assessed with immunohistochemistry (IHC), Her2 status with IHC and fluorescence in situ hybridization.

**Results:**

ER/PR were positive in 74–79% cases, ER/PR/Her2 negative in 6.16–10.70% and Her2 positive in 11.49–13.88%/year. Ki67 had median values as 15–17.5% in ER/PR-positive cases, 55–60% in triple-negative cases and 30–32.50% in Her2-positive cases. Histological grading distribution for well (G1), moderately (G2) and poorly (G3) differentiated carcinomas was 15.8–19.1% for G1, 54.2–54.8% for G2 and 21.7–23.7% for G3 cases. Variation in yearly distributions was not significant in any of these markers.

**Conclusions:**

Predictive markers displayed a yearly similar distribution in breast cancer cases independently of grading or of intrinsic subtypes. These results point to a qualitative high performance of predictive marker assessment in breast cancer, corresponding to expected on average positivity rate per marker and per year. It is recommended to monitor positivity rate of ER, PR, Ki67 and Her2 yearly or periodically to comply with quality assurance requirements.

## Introduction

Breast cancer is the most common invasive cancer and the leading cause of cancer-related deaths in women worldwide (McGuire et al. 2015). One of the main pillars of breast cancer management is targeted therapy (Slamon et al. 2011). There is a wide range of biomarkers being expressed by different breast cancer subtypes. Targeted therapy is mainly planned and based on the profile of biomarker expression, which basically comprise ER/PR Her2 and Ki67 Labelling Index (LI) (Hicks and Tubbs 2005).

The link between HER2 overexpression/amplification and breast cancer growth and development has been an important hallmark in targeted breast cancer therapy (Slamon et al. 1987). HER2 is a transmembrane tyrosine kinase receptor expressed in approximately 20% of invasive breast cancer, accounting for aggressive phenotypes, early metastasis and lower rate of disease-free and overall survival (Slamon et al. 2011; Vogel 2010). Anti-HER2 therapy, such as trastuzumab and lapatinib, specifically bind to the extracellular domain of the receptor, inhibiting tumor cell growth and inducing cell apoptosis (Slamon et al. 2011; Dowsett et al. 2000).

Furthermore, estrogen (ER) and progesterone (PR) receptors are important mediators in breast cancer growth by acting as ligands on the ER and PR expressed on two-thirds of all breast cancers (Duffy et al. 2017). For decades, ER has been the most important biomarker and crucial for determining a patient’s eligibility for endocrine therapy (Duffy et al. 2017; Hammond et al. 2010). Although PR could not be proven to be an equally potent predictive factor, it is still frequently measured alongside (Hammond et al. 2010).

An additional biomarker is Ki67, a protein expressed in all cells during proliferation phase, giving insight into proliferation activity of any cell population (Scholzen and Gerdes 2000; Tashima et al. 2015). A high rate of Ki67 is associated with tumor growth, higher tumor grades, earlier metastasis and poor disease outcome (Scholzen and Gerdes 2000; Tashima et al. 2015). Since Ki67 indicates tumor growth activity, assessment of Ki67 can be used to estimate tumor response to therapies that specifically target dividing cells, such as chemotherapy in particular (Scholzen and Gerdes 2000; Tashima et al. 2015). Despite low cost and wide availability, Ki67 assessment is not yet fully established in routine breast cancer diagnostics, due to methodological problems in determination and unclear clinical cutoffs (Hicks and Tubbs 2005; Curigliano et al. 2017). To include Ki67 into routine clinical diagnostics, international standardization of cutoff values as well as staining procedures and pathological validation are needed (Scholzen and Gerdes 2000; Curigliano et al. 2017).

Biomarkers are most commonly assessed by immunohistochemistry (IHC) and in situ hybridization technologies labeled with fluorescence, silver or chromogenic substances [fluorescence in situ hybridization (FISH), SISH, CISH], respectively (Hicks and Tubbs 2005; Harvey et al. 1999).

Immunohistochemistry is used widely in routine diagnostics, due to its low cost and availability (Ghaffari et al. 2011). However, this technique depends strongly on pre-analytical factors, such as duration of fixation and analytic factors such as choice of anti-HER2 antibodies, lowering reproducibility and accuracy of the results.

FISH is a technique that detects the presence or absence of a DNA sequence in a cell (Ghaffari et al. 2011). This method has high specificity and sensitivity; however, it requires not only expertise in signal interpretation but also specialized laboratory facilities (Sui et al. 2009). Therapy planning depends on the profile of biomarkers expressed by the given breast cancer (Duffy et al. 2017). Effectiveness of targeted therapy relies on accuracy of diagnostics. A high rate in false-positive and false-negative results, respectively, can lead to wrong choice of therapy, generating excess expenses and depriving patients from receiving appropriate care (Hicks and Tubbs 2005; Choritz et al. 2011; Varga et al. 2013).

There are little data on accuracy and comparability of the collected data among institutes, or even within one single institute over a certain period of time (Choritz et al. 2011; Varga et al. 2013). A constant rate of biomarkers over the given period of time and the concordance of the measured positivity rates with rates of other institutions serve as an index for good quality (Varga et al. 2013; Cserni et al. 2014; Rüschoff et al. 2017).

The aim of this retrospective study is to assess the variation and accuracy of predictive markers as ER, PR, Her2 status and Ki67 measured by FISH and IHC assessment in routine diagnostics in one single institution in the period 2015–2018. The goal is to contribute to standardization of assessments and the establishment of guidelines on how the assessments are to be performed and which results are to be expected.

## Materials and methods

Consecutive original pathology reports with invasive breast cancer from the Institute of Pathology and Molecular Pathology, University Hospital Zurich Switzerland between 2015 and 2018 were analyzed. Altogether 2214 consecutive breast cancer cases were included in the analysis. Data on ER, PR, Her2 status and Ki67 were available in all cases.

Most information was available on preoperative breast core and vacuum-assisted biopsies. Further data (in case of re-testing at triple negative, Her2 equivocal cases and re-testing of Ki67 LI) were retrieved also from the surgical specimens.

Negative hormone receptors were re-assessed on the surgical specimens. Exceptions occurred in cases of complete pathological response after neoadjuvant chemotherapy, and in these instances, the hormone receptor status was retrieved from the biopsies. Her2 status was re-assessed on surgical specimens at equivocal cases (score 2+) on biopsies and/or newly diagnosed high-grade tumor component (G3) in surgical specimens.

Histological grading for the study was retrieved from the definitive surgical specimens, with exceptions of complete pathological response after neoadjuvant chemotherapy or if the surgical specimen was examined in an external pathology unit elsewhere.

In total, 1899 core and/or vacuum biopsies and 1300 surgical specimens from altogether 2214 consecutive patients were enrolled into the study.

All reactions were performed on formalin fixed paraffin-embedded samples during routine diagnostic procedure. No further analyses were done for this study outside of the routine diagnostic procedure.

### Immunohistochemistry (IHC) for HER2 status, ER, PR and Ki67 and fluorescence in situ hybridization for HER2 status

Assessments were performed in the same way as published previously according to the standard diagnostic procedures of the Institute of Pathology and Molecular Pathology University Hospital Zurich (Varga et al. 2013, 2015).

### Guidelines to evaluate scoring

ER, PR, and Her2 status were evaluated using the time current ASCO/CAP guidelines, and Ki67 was assessed with visual assessment per eyeballing as described earlier based on the SAKK 28/12 study (Hammond et al. 2010; Choritz et al. 2011; Varga et al. 2015; Wolff et al. 2013, 2018; Burstein et al. 2010; Curigliano et al. 2017).

Cases that were FISH positive or showed a 3+ IHC score were considered HER2 positive. Cases that were HER2 negative in FISH assay or lower than 3+ in IHC and had no hormone receptor expression were considered triple negative. Cases that had an ER and/or PR expression rate above 1% were considered hormone receptor positive.

### Statistics/interpretation of results

Measurements obtained in each year over this period were compared with each other. A steady rate over a certain period of time accounts for assessment quality and was the primary endpoint of this study.

The rates of HER2 assessed with IHC and FISH were evaluated for each year in the period 2015–2018 and compared among each other and with the guidelines. To objectify the concordance of IHC and FISH measurements of HER2, the Spearman correlation and Fisher exact test were used.

Frequency of Ki67 (MIB-1) in HER2, PR and ER-positive and triple-negative cases as well as in all luminal subtypes, was assessed and visualized using frequency distribution histograms. PR and ER positive included all individuals that had a receptor rate above 1%. To exclude significant yearly variation of data and to verify the concordance of the results with current guidelines, the means of Ki67 (MIB-1) were compared using the Kruskal–Wallis test.

Unless otherwise noted, results are expressed as mean ± standard deviation (SD). For all statistical analysis and figures, GraphPad Prism 5.0 for Windows (GraphPad Software, San Diego, USA) was used. Statistical significance was determined with a *p* value < 0.05 and a confidence interval of 95%.

### Ethical approval

This study was designed and conducted as a quality control study of the institute and was approved by the Ethical Committee of Zurich (KEK-2012-0553).

## Results

### Her2 status

**Fig. 1:**
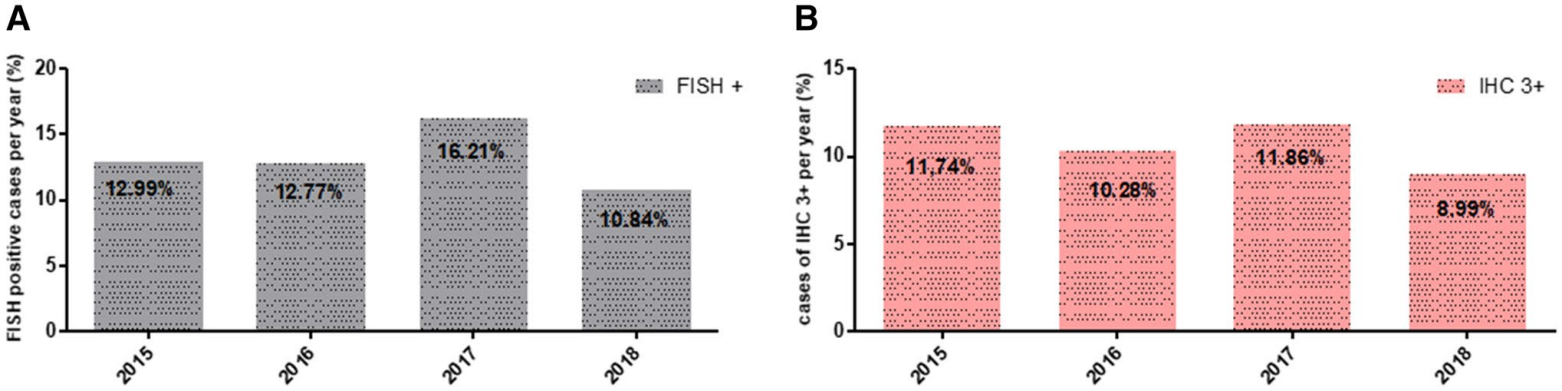
**a** Her2 FISH positivity rate per year.** b** Her2 IHC 3+ rate per year. *FISH* fluorescence in situ hybridization, *IHC* immunohistochemistry

Detailed results are shown in Table 1 and in Fig. 1.

**Table 1:** Concordance of IHC and FISH in assessment of HER2 status in routine diagnostic of breast cancer in the years 2015, 2016, 2017, and 2018

Her2 status IHC/FISH	0	1+	2+	3+
2015				
FISH amplified	0 (0%)	3 (1 5%)	9 (8 6%)	61 (92 4%)
Non-amplified	199 (100%)	190 (98 5%)	95 (91 4%)	5 (7 6%)
Total (*n* = 562)	199	193	104	66
2016				
FISH amplified	1 (0 4%)	2 (1%)	14 (14%)	60 (96 8%)
Non-amplified	240 (99 6%)	199 (99%)	85 (86%)	2 (3 2%)
Total (*n* = 603)	241	201	99	62
2017				
FISH amplified	1 (0 4%)	6 (2 5%)	22 (20 6%)	79 (100%)
Non-amplified	239 (99 6%)	234 (97 5%)	85 (79 4%)	0 (0%)
Total (*n* = 666)	240	240	107	79
2018				
FISH amplified	1 (0 7%)	3 (2 1%)	5 (8 8%)	35 (100%)
Non-amplified	147 (99 3%)	140 (97 9%)	52 (91 2%)	0 (0%)
Total (*n* = 383)	148	143	57	35

*IHC* immunohistochemistry, *FISH* fluorescence in situ hybridization

#### 2015–2018

FISH Her2 positivity rate showed a slight variation in these 4 years, varying between 10.8 and 16.21% per year. IHC Her2 score 3+ frequency was similar and varied between 8.99 and 11.86% per year. Differences between the years both in FISH testing and IHC assessments were statistically not significant and were considered as stable status.

The Chi square statistic is 0.0565. The *p* value is 0.996491. The result is not significant at *p* < 0.05.

As to concordance between IHC and FISH measurements, all years had a concordance between IHC and FISH tests > 95%. This calculation included the IHC 0/1+ category with FISH amplification and IHC score 3+ category without FISH amplification.

### Ki67 per intrinsic subtype

Here, below a short summary of each year is described.

**Fig. 2:**
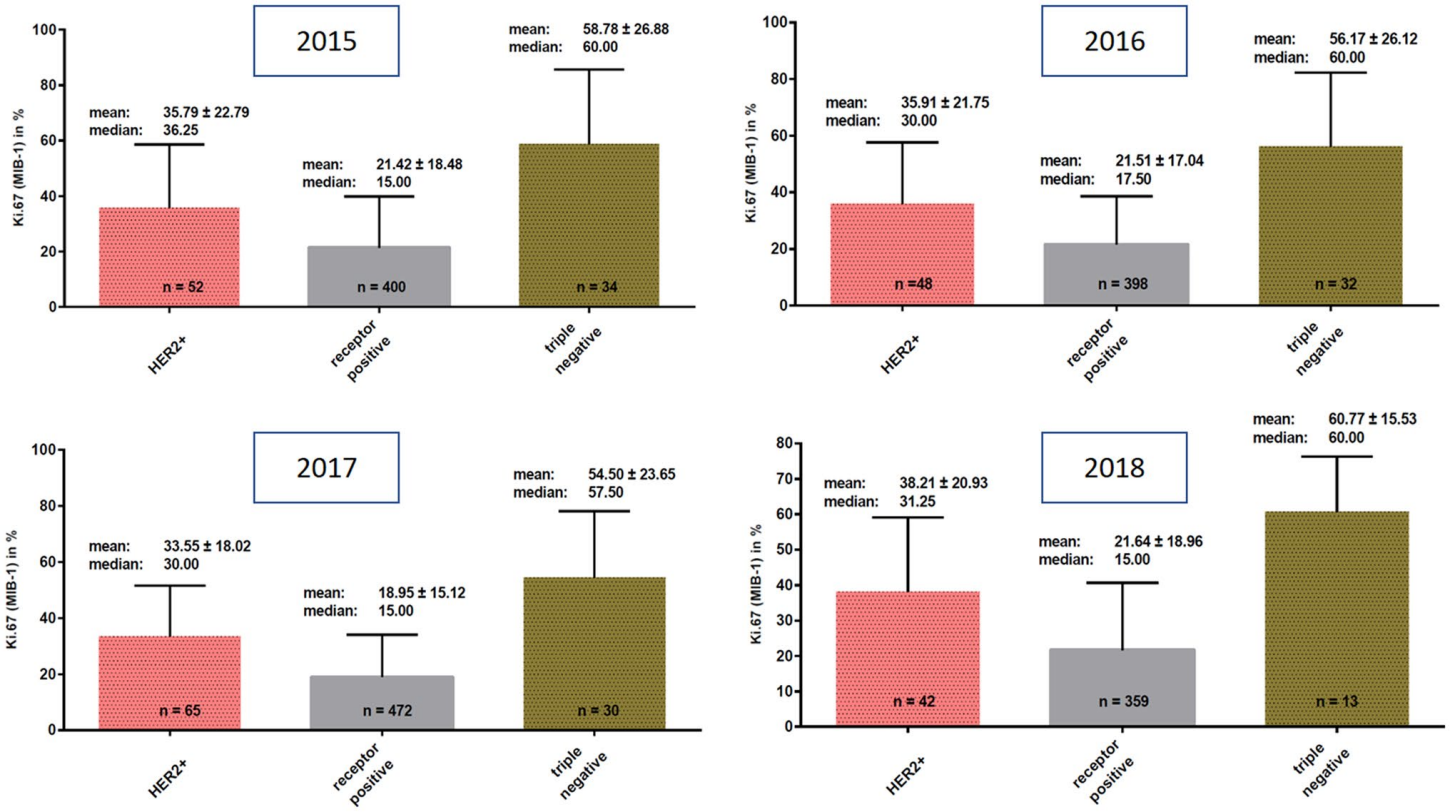
Mean and median Ki67 in HER2-positive cases, hormone receptor-positive cases and in triple-negative cases derived from core biopsies and surgical specimen combined in the years 2015, 2016, 2017, and 2018

**Fig. 3:**
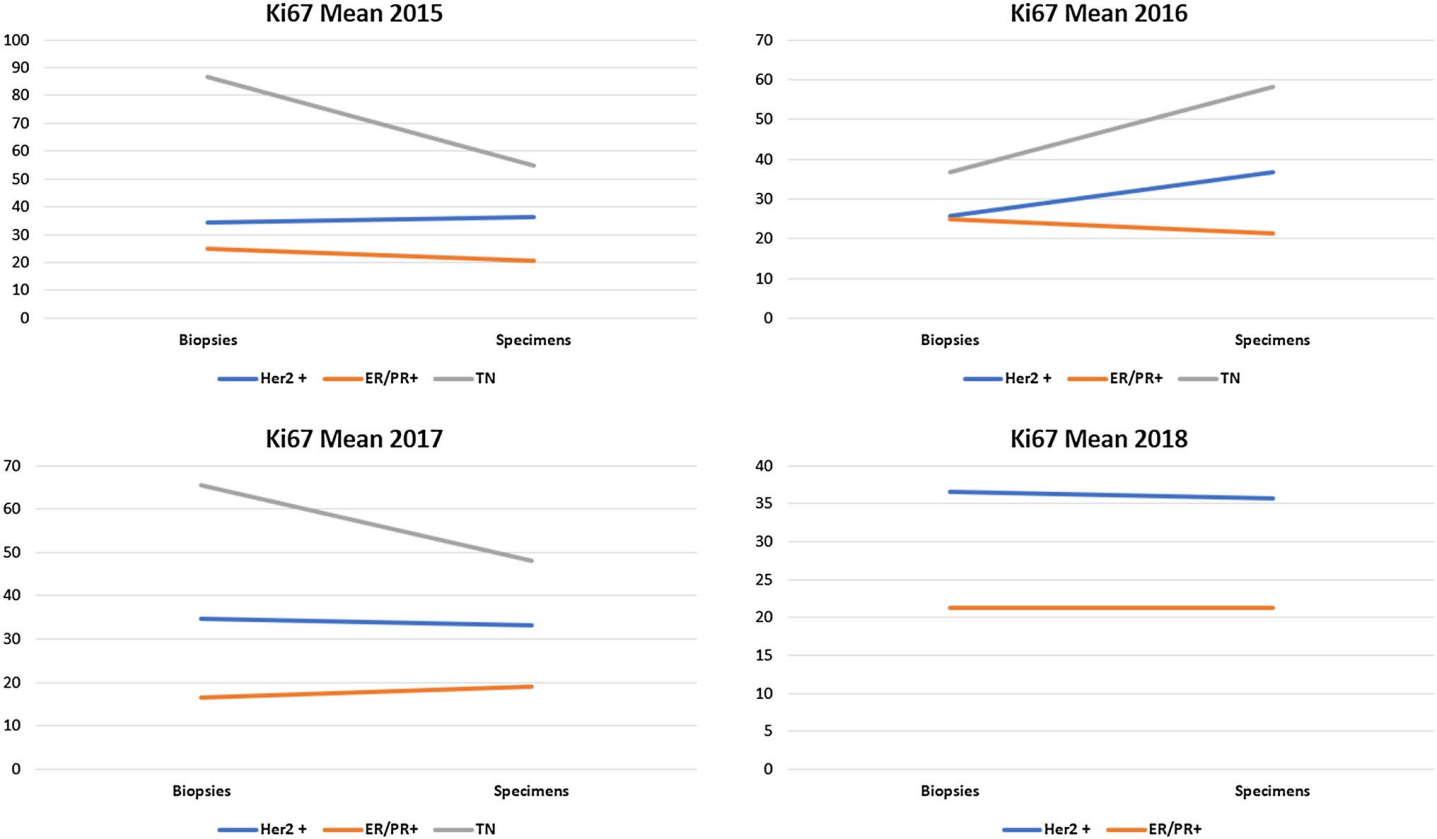
Mean Ki67 distribution in preoperative biopsies and in surgical specimens stratified according to ER2-positive cases, hormone receptor-positive cases and in triple-negative cases derived from core biopsies and surgical specimen combined in the years 2015, 2016, 2017, and 2018

Detailed results are shown in Table 2 and in Figs. 2 and 3.

**Table 2:** Mean and median Ki67 (MIB-1) values per year for intrinsic subtypes derived combined from core/vacuum biopsies and surgical specimen, from core/vacuum biopsies only, and from surgical specimens only

Ki67	HER2 positive	ER or PR positive	Triple negative
*Core/vacuum biopsies and surgical specimen combined*			
2015			
Mean	35.79% ± 22.79%	21.42% ± 18.48%	58.78% ± 26.88%
Median	36.25% *n* = 52	15.00% *n* = 400	60.00% *n* = 34
2016			
Mean	35.91% ± 21.75%	21.51% ± 17.04%	56.17% ± 26.12%
Median	30.00% *n* = 48	17.50% *n* = 398	60.00% *n* = 32
2017			
Mean	33.55% ± 18.02%	18.95% ± 15.12%	54.50% ± 23.65%
Median	30.00% *n* = 65	15.00% *n* = 472	57.50% *n* = 30
2018			
Mean	38.21% ± 20.93%	21.67% ± 18.96%	60.77% ± 15.53%
Median	31.25% *n* = 42	15.00% *n* = 359	60.00% *n* = 14
*Core/vacuum biopsies only*			
2015			
Mean	34.29% ± 21.45%	24.98% ± 21.26%	66.56% ± 28.55%
Median	30.00% *n* = 17	20.00% *n* = 82	90.00% *n*n= 8
2016			
Mean	25.83% ± 5.774%	24.80% ± 18.47%	36.67% ± 23.63%
Median	22.50% *n* = 3	17.50% *n* = 32	45.00% *n* = 3
2017			
Mean	34.65% ± 17.72%	16.38% ± 13.29%	65.45% ± 16.95%
Median	30.00% *n* = 17	10.00% *n* = 98	70.00% *n* = 11
2018			
Mean	38.71% ± 20.87%	21.70% ± 18.91%	60.77% ± 15.53%
Median	35.00% *n*= 35	15.00% *n* = 325	60.00% *n* = 13

Ki67 (MIB1)	HER2 positive	ER or PR positive	Triple negative
Surgical specimens only			
2015			
Mean	36.51% ± 23.68%	20.50% ± 17.61%	56.38% ± 26.47%
Median	40.00% *n* = 35	15.00% *n* = 318	50.00% *n* = 26
2016			
Mean	36.58% ± 22.28%	21.23% ± 16.91%	58.19% ± 25.89%
Median	30.00% *n* = 45	17.50% *n* = 366	60.00% *n* = 28
2017			
Mean	33.16% ± 18.29%	18.95% ± 15.19%	48.16% ± 25.02%
Median	26.25% *n* = 48	15.00% *n* = 374	50.00% *n* = 19
2018			
Mean	35.71% ± 22.76%	21.32% ± 19.72%	–
Median	30.00% *n* = 7	15.00% *n* = 34	– *n* = 0

Ki67 was assessed as combined value on core/vacuum biopsies and also in surgical specimens and also separate to intrinsic subtype as ER/PR positive, Her2 positive and triple negative. We found significant differences of Ki67 values between the intrinsic subtypes with almost similar results per year. However, the mean and median values of each intrinsic subtype remained constant over the 4 years, and the differences were statistically not significant. The results whether Ki67 value was taken from core/vacuum biopsies and/or from surgical specimens were comparable with each other and were statistically not significant.

#### 2015

*Combined from core biopsies and surgical specimen* Mean Ki67 was 35.79% ± 22.79% in HER2-positive cases, 21.42% ± 18.48% in hormone receptor-positive cases and 58.78% ± 26.88% in triple-negative cases, and these differences were statistically significant (*p* < 0.0001, *p* < 0.05).

*From core/vacuum biopsies only* Mean Ki67 was 34.29% ± 21.45% in HER2-positive cases, 24.98% ± 21.26% in hormone receptor-positive cases and 66.56% ± 28.55% in triple-negative cases.

These differences were statistically significant between Her2 and ER/PR-positive cases (*p* < 0.01).

*From surgical specimens only* Mean Ki67 was 36.51% ± 23.68% in HER2-positive cases, 20.50% ± 17.61% in hormone receptor-positive cases, and 56.38% ± 26.47 in triple-negative cases.

Differences between HER2-positive and hormone receptor-positive cases was significant (*p* < 0.001).

#### 2016

*Combined from core/vacuum biopsies and surgical specimen* Mean Ki67 was 35.91% ± 21.75% in HER2-positive cases, 21.51% ± 17.04% in receptor-positive cases and 56.17% ± 26.12% in triple-negative cases.

Difference between the three intrinsic subtypes was statistically significant (*p* < 0.0001).

*From core/vacuum biopsies only* Mean Ki67 in HER2-positive cases was 25.83% ± 5.774%, in receptor-positive cases 24.80% ± 17.50% and in triple-negative cases 36.67% ± 23.63%. These differences were statistically not significant.

*From surgical specimens only* Mean Ki67 values were 36.58% ± 22.28% in HER2-positive cases, 21.23% ± 16.91% in hormone receptor-positive cases and 58.19% ± 25.89% in triple-negative cases.

The difference between the three groups was statistically significant (*p* < 0.0001).

#### 2017

*Combined from core/vacuum biopsies and surgical specimen* Mean Ki67 in HER2-positive cases was 33.55% ± 18.02%, in receptor-positive cases 18.95% ± 15.12% and in triple-negative cases 54.50% ± 23.65%.

The differences were statistically significant (*p* < 0.0001, *p* < 0.05).

*From core/vacuum biopsies only* Mean Ki67 in HER2-positive cases was 34.65% ± 17.72%, in hormone receptor-positive cases 16.38% ± 13.29%, in triple-negative cases 65.45% ± 16.95%.

Difference between HER2-positive and hormone receptor-positive cases was significant (*p* < 0.001).

*From surgical specimens only* Mean Ki67 in HER2-positive cases was 33.16% ± 18.29%, in hormone receptor-positive cases 18.95% ± 15.19% and in triple-negative cases 48.16% ± 25.02%.

The differences were statistically significant (*p* < 0.001).

#### 2018

*Combined from core/vacuum biopsies and surgical specimen* Mean Ki67 in HER2-positive cases was 38.21% ± 20.93%, in hormone receptor-positive cases 21.67% ± 18.96% and in triple-negative cases 60.77% ± 15.53%.

Difference between HER2 positive and hormone receptor-positive cases was statistically significant (*p* < 0.0001).

*From core/vacuum biopsies only* Mean Ki67 in HER2-positive cases was 38.71% ± 20.87%, in hormone receptor-positive cases 21.70% ± 18.91% and in triple-negative cases 60.77% ± 15.53%.

The differences were statistically significant (*p* < 0.001, *p* < 0.0001).

*From surgical specimens only* Mean Ki67 values were 35.71% ± 22.76% in HER2-positive cases and 21.32% ± 19.72% in hormone receptor-positive cases. None of the surgical specimens in 2018 belonged to the triple-negative subtype.

Differences between HER2 and hormone receptor-positive cases were statistically significant (*p* < 0.001).

#### Yearly mean Ki67 variation in HER2-positive patients

The mean Ki67 in all HER2-positive patients assessed in the period 2015–2018 showed no significant yearly variation in mean values (mean Ki67 in HER2-positive cases in 2015: 35.79% ± 22.79%, vs. 2016: 35.91% ± 21.75%, vs. 2017: 33.55% ± 18.02%, vs. 2018: 38.21% ± 20.93%, *p* = 0.74).

#### Yearly mean Ki67 in hormone receptor-positive patients

The means of Ki67 in hormone receptor-positive cases assessed in the period 2015–2018 showed no significant yearly variation (mean Ki67 in hormone receptor-positive cases in 2015: 21.42% ± 18.48%, vs. 2016: 21.51% ± 17.04%, vs. 2017: 18.95% ± 15.12%, vs. 2018: 21.67% ± 18.96%, *p* = 0.26).

#### Yearly mean Ki67 in triple-negative patients

The means of Ki67 in all triple-negative cases assessed in the period 2015–2018 showed no significant yearly variation (mean Ki67 in triple-negative cases in 2015: 58.78% ± 26.88%, vs. 2016: 56.17% ± 26.12%, vs. 2017: 54.50% ± 25.03%, vs. 2018: 60.77% ± 15.53%).

#### Concordance between Ki67 values obtained in preoperative core biopsies and surgical specimens (Fig. 3)

The highest level if discordant Ki67 values were obtained in the triple-negative group with variations in both directions HER2 and ER/PR-positive cases remained relatively stable in both type of tissues.

### Hormone receptors

Hormone receptors showed a very similar distribution in all combinations (ER pos/neg, PR pos/neg) over the 4-year period. ER and PR were both positive in 73.84%, 74.96%, 78.23% and 76.34% and both negative in 13.17%, 13.27%, 9.31%, and 6.79%.

The distribution of intrinsic subtypes as hormone receptor positive, HER2 positive and triple negative remained constant in the distribution over the 4-year period.

**Fig. 4:**
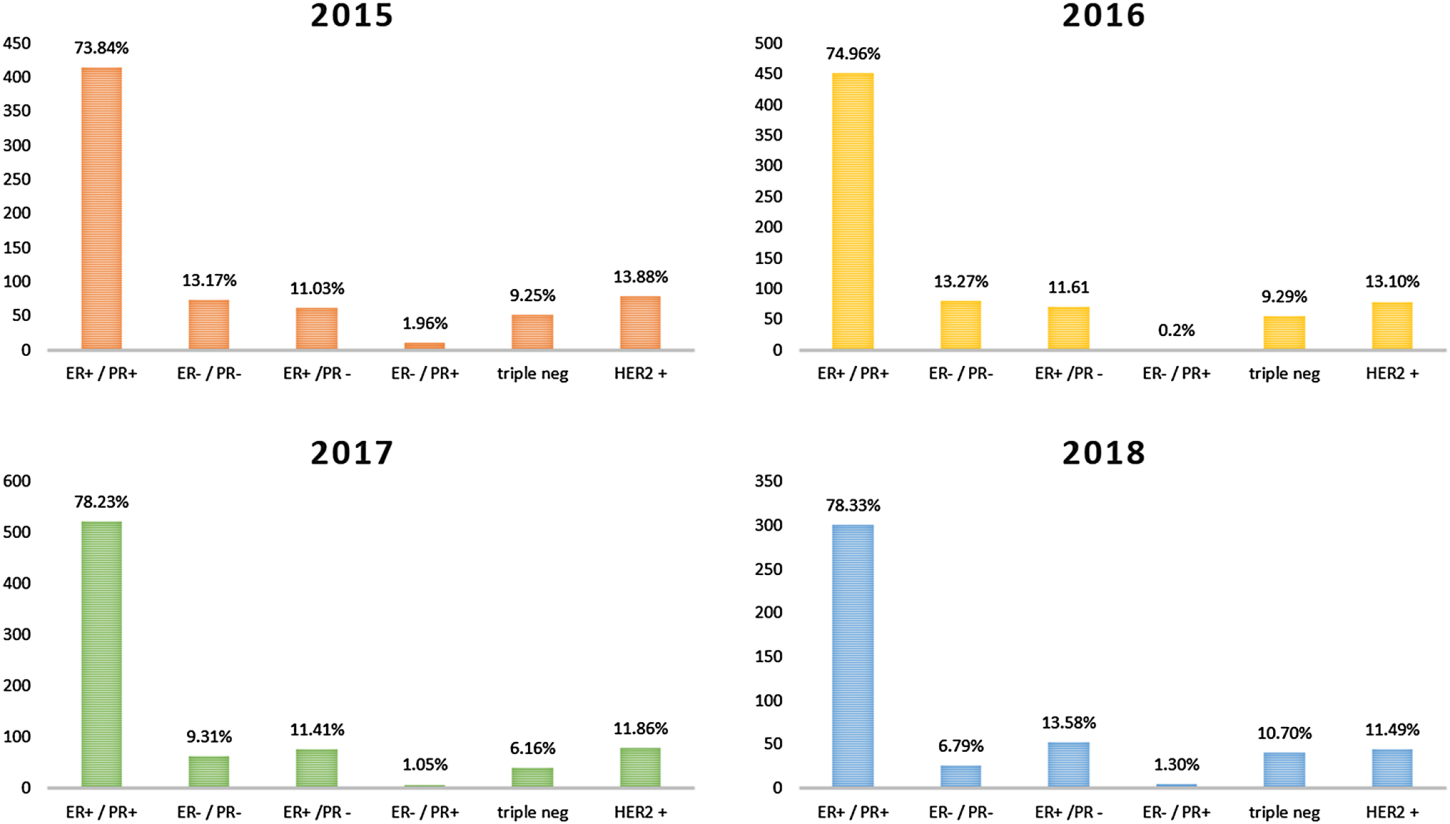
Hormone receptor status frequency per year distributed according to intrinsic subtypes.** a** 2015,** b** 2016,** c** 2017 and** d** 2018

Detailed results are shown in Table 3 and in Fig. 4.

**Table 3:** Hormone receptor status and intrinsic subtype frequency per year

	ER+/PR+	ER−/PR−	ER+/PR−	ER−/PR+
2015 (*n* = 562)	73.84% (415)	13.17% (74)	11.03% (62)	1.96% (11)
2016 (*n* = 603)	74.96% (452)	13.27% (80)	11.61% (70)	0.2% (1)
2017 (*n* = 666)	78.23% (521)	9.31% (62)	11.41% (76)	1.05% (7)
2018 (*n* = 383)	78.33% (300)	6.79% (26)	13.58% (52)	1.3% (5)
Total mean	76.34%	10.63%	11.91%	1.13%

	ER positive	PR positive	Triple negative	HER2 positive
2015 (*n* = 562)	84.70% (476)	75.80% (426)	9.25% (52)	13.88% (78)
2016 (*n* = 603)	86.40% (521)	75.12% (453)	9.29% (56)	13.10% (79)
2017 (*n* = 666)	89.64% (597)	79.28% (528)	6.16% (41)	11.86% (79)
2018 (*n* = 383)	91.91% (352)	79.63% (305)	10.70% (41)	11.49% (44)
Total mean	88.16%	77.46%	8.85%	12.58%

### Histological grading

The distribution of histological grade as well (G1), moderately (G2) and poorly (G3) differentiated invasive breast carcinomas remained almost identical over the 4-year period only with minimal variation among the grades. Grad1 was between 15 and 19.1%, G2 between 54.2 and 55.4% and G3 between 21.7 and 23.8%.

**Fig. 5:**
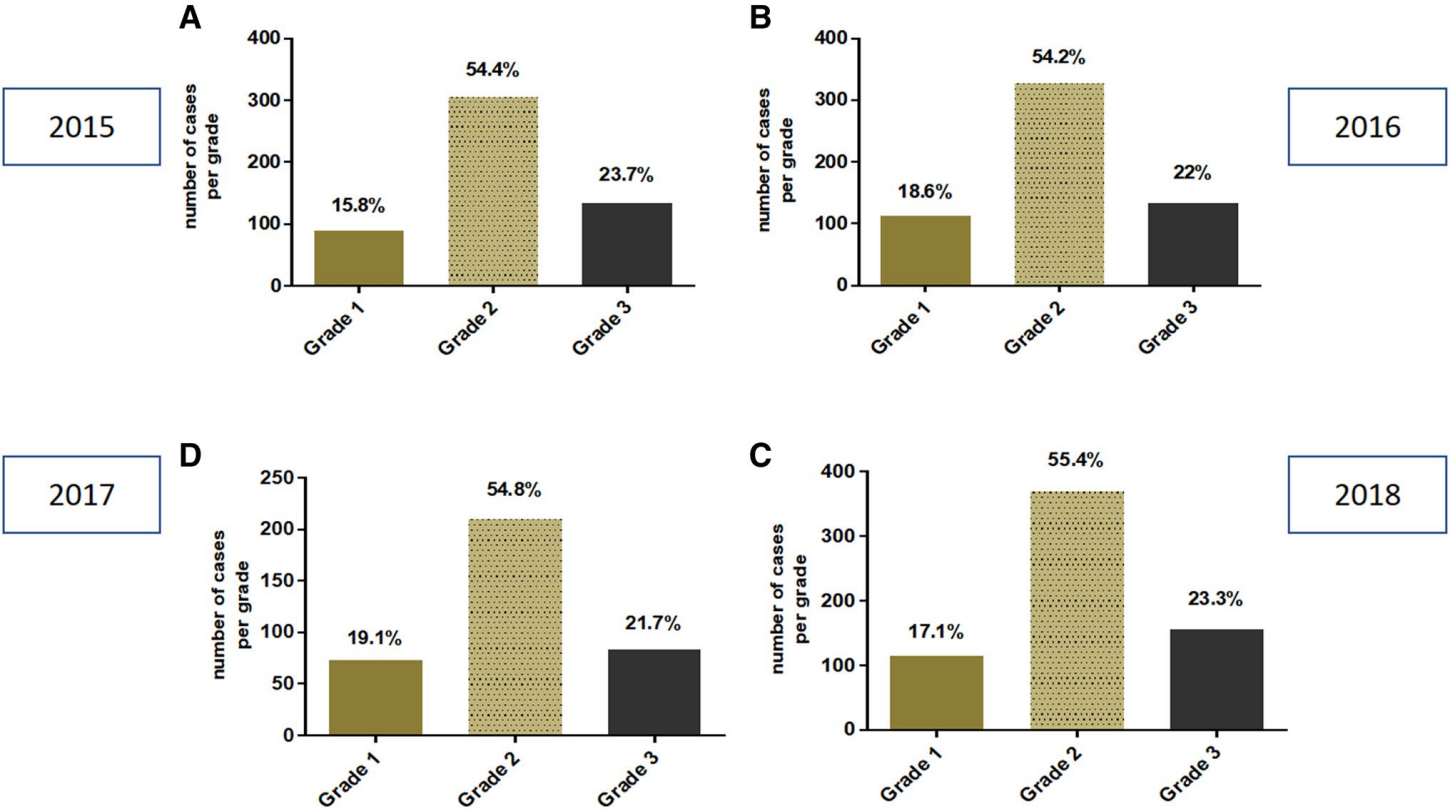
Frequency of histological grades derived in 2015 (**a**), 2016 (**b**), 2017 (**c**) and 2018 (**d**)

Detailed results are shown in Table 4 and in Fig. 5.

**Table 4:** Frequency of histological grades derived in 2015, 2016, 2017 and 2018

	Grade 1	Grade 2	Grade 3
2015 (*n* = 562)	89 (15.8%)	306 (54.4%)	133 (23.7%)
2016 (*n* = 603)	112 (18.6%)	327 (54.2%)	133 (22%)
2017 (*n* = 666)	114 (17.1%)	369 8 (55.4%)	155 (23.3%)
2018 (*n* = 383)	73 (19.06%)	210 (54.8%)	83 (21.7%)
Total mean	17.64%	54.7%	22.67%

## Discussion

In this study, we performed a quality control study on the yearly distribution of predictive factors in breast cancer as ER/PR, HER2 and KI67. By generating evidence-based data on biomarker assessments, this study aims to enhance matters in standardization of intrinsic subtype determination.

Accuracy of intrinsic subtype determination is crucial for identifying patients eligible for a particular therapy, thereby providing appropriate care and minimizing side effect exposure as well as costs incurred by inadequate therapy. Proficiency and compliance to the current ASCO-CAP guidelines of an institute as well as intra- and inter-institutional concordance of results have to be reviewed frequently to assure quality of assessments (Hammond et al. 2010; Varga et al. 2013; Wolff et al. 2018; Bilous et al. 2003; Romain et al. 1995). To monitor accuracy of our institution, stability of HER2 positivity rates and concordance rates of HER2 assessments with FISH and IHC, respectively, over a certain period of time served as indices for quality of assessments. For reliability of ER and PR assessments, the ASCO-CAP guidelines recommend that the yearly hormone receptor positivity rate is documented by each institute (Hammond et al. 2010). There is no clear recommendation by the ASCO-CAP guidelines concerning the Ki67 cutoffs, yet they have to be clarified (Wolff et al. 2018; Bilous et al. 2003; de Azambuja et al. 2007).

Regarding HER2 status, this study shows that concordance rates of FISH and IHC assessments of HER2 have significantly improved compared to earlier reports among other from the same institution (Varga et al. 2013). In 2015–2018, concordance rate of HER2 measured by IHC and FISH was above the 95% rate recommended by the ASCO-CAP guidelines (Wolff et al. 2013). Further, showing a significant improvement compared with the concordance rates observed in 2001–2004 and 2007–2011 in the same institution with reported improvement of overall concordance between IHC and FISH from 84% 2001–2004 to 97% in 2011–2012 (Varga et al. 2013).

Reasons for discordance between IHC and FISH assessments, which were only a few cases in these 4-year period, are not entirely clear. Possible reasons for this discordance are most commonly false-positive IHC 2+ interpretations, focal HER2 positivity expressed only in one single biopsy specimen or not DNA-coupled synthesis of the HER2 protein.

HER2 positivity rates remained stable, showing no significant yearly variation in the observed period and were further concordant with rates reported in the previous literature (Ghaffari et al. 2011; Choritz et al. 2011; Bilous et al. 2003). In the years 2015–2018, we observed a mean FISH HER2 positivity rate of 13.2% (range 10–17%). Both FISH and IHC measured HER2 positivity rates dropped slightly in 2018, possibly due to application of the new ASCO-CAP guideline recommendations published 2018. As was previously reported within the same institute between 2005 and 2010, a mean FISH HER2 positivity rate of 15.8% (range 13–19%) had a slight drop to 13–14% after implementation of the modified ASCO guidelines (Varga et al. 2013; Varga and Noske 2015). An interesting observation that might require further investigation was that the mean HER2 positivity rates in 2015, 2016 and 2018 where higher when both IHC and FISH were combined, than the results of each of the two methods assessed separately.

We analyzed and compared mean Ki67-Proliferation Index for the following three subtypes: HER2+, ER or PR positive and triple negative. The combined results for core biopsies and surgical specimen showed significant difference in mean Ki67-proliferation among these subtypes. Whereas steroid receptor-positive subtypes are associated with better prognosis, showing less adverse outcome and higher overall survival rate (Makretsov et al. 2011). Correspondingly, higher values of Ki67 are also associated with more adverse outcomes (Tashima et al. 2015; de Azambuja et al. 2007). However, there was neither significant yearly variation of mean nor median Ki67 for neither of the subtypes, which is a reliable quality assurance result for constant performance.

The differences in Ki67 values in different intrinsic subtypes have been also reported in the literature, as Cserni et al. published 33.3% mean and 30% median Ki67 in HER2-positive cases. Tashima et al. did not publish Ki67 values for HER2-positive cases, but 43% mean and 41% median Ki67 in HER2-enriched cases instead (Tashima et al. 2015; Cserni et al. 2014), these results are very similar to the Ki67 values obtained in our study.

Regarding Ki67 values in triple-negative cases, data in our study are very similar to the reported literature data (Tashima et al. 2015; Cserni et al. 2014).

When core biopsies and surgical specimen were observed separately, there was a slight deviation from the results in both directions possibly due to a sample size bias and to intratumoral heterogeneity.

Continuous distribution and various pre-analytical and analytical factors influence accuracy of Ki67 assessment and impede establishment of official cutoffs (Duffy et al. 2017; Scholzen and Gerdes 2000). During the St. Gallen International Expert Consensus Conference in 2017, caution was recommend as reproducibility issues of Ki67 assessments are still unresolved (Curigliano et al. 2017). Internal standardization in terms of defining own laboratory values per given institution can be a possible way of standardization even though the own laboratory values may considerably vary (Curigliano et al. 2017). Our results were concordant with the numbers published by Cserni et al. (2014) and Tashima et al. (2015). Interestingly, Cserni et al. came up with almost identical values.

Hormone receptor-positive cases were quite concordant with previously published and expected results. Cserni et al. published 18.5% mean for ER-positive cases, 18.3% mean for PR positive and 15% median Ki67 for both ER and PR-positive cases. Meanwhile, Tashima et al. published 23.2% mean and 19% median Ki67 for luminal HER2-negative cases (Tashima et al. 2015; Cserni et al. 2014).

As the first EORTC report on steroid receptor distribution postulates, a comparable positivity rate over time and inter-laboratory is an index for assessment quality (Romain et al. 1995). Hence, distribution of steroid receptors in the patient population of an institute has to be monitored frequently (Romain et al. 1995; Rhodes et al. 2000). Hormone receptor status in our institute showed no significant yearly variation. ER-positive cases ranged from 85 to 92% with a mean of 88.16%, while PR-positive cases ranged from 76 to 80% with a mean of 80.43%. In comparison, Rhodes et al. documented in 2000 a mean ER positivity rate of 76.9% and 70.6% in institutes of high assessment sensitivity and low sensibility, respectively, and a mean PR positivity rate of 62.8% and 51.4% in institutes of high sensibility and low sensibility, respectively (Rhodes et al. 2000). Two-thirds of breast cancer are supposed to be ER positive this accounts for roughly 75–80%. Thus, our measurements fall into the expected range (Vohra et al. 2016; Gruvberger et al. 2001).

We observed a frequency and distribution of the four different ER/PR status, namely ER+/PR+, ER−/PR−, ER+/PR− and ER−/PR+. Our results were concordant, with the publication of Kenneth et al. who investigated the distribution of steroid receptor in breast cancer throughout different ethnic groups in 2001 (Chu et al. 2001).

Significance and even the existence of the four ER/PR status is controversial and not entirely proven (Chu et al. 2001). The ER−/PR+ status is thought to be the result of false-negative ER measurement and the frequency of ER+/PR− is expected to be very low (Vohra et al. 2016; Chu et al. 2001). Our results showed were hence concordant with the previous literature.

Regarding histological grading, there was a clear majority of grade 2 breast cancers with more than 50% of grade 2 breast cancer cases in the entire period 2015–2018. This tendency is not entirely clear and not completely concordant with the rates published at Nottingham University by Rakha et al. in 2008, where grade 1 accounted for 18.6%, grade 2 for 35.6% and grade 3 for 45.6% of breast cancer cases (Rakha et al. 2008). However, in 2009, Rakha et al. observed tumors with mixed ductal and lobular features publishing the following distribution: 13% grade 1, 82% grade 2 and 44% grade 3 (Rakha et al. 2009), which is very similar to our results. Further investigation on reasons for this discrepancy of grade frequency would be of interest.

## Conclusions

Standardization and quality control of biomarker assessment have been a matter of discussion ever since biomarkers have started to play a role in breast cancer management. The aim is to achieve optimal inter- and intra-laboratory concordance and stability of amplification rates. Frequent monitoring of assessment quality is, therefore, crucial. In this study, we gathered data on amplification rate stability over the period of 4 years and concordance in HER2 testing with FISH and IHC. By showing an improvement of results compared to previous investigations at our institute, we demonstrated that compliance to ASCO-CAP guidelines has a positive effect on amplification rate stability and concordance of IHC and FISH technique and hence accuracy of assessments. Additionally, we could show a constant yearly performance on KI67 both on preoperative biopsies and surgical specimens, which was the same on hormone receptor status and on histological grading. Periodical monitoring predictive factors in breast cancer is essential to keep a high quality and reliable performance in routine pathological diagnostic service.

## Abbreviations

ER: Estrogen receptor
FISH: Fluorescent in situ hybridization
HER2: Human epidermal growth factor
IHC: Immunohistochemistry
PR: Progesterone receptor
